# Supplementary figures and images for: Highly Sensitive Detection of miR-200c in Metastatic Lymph Nodes Using Scanning Single-Molecule Counting
Source: Cancers (Basel). 2025 Sep 26;17(19):3133. doi: 10.3390/cancers17193133 (PMC12523761; doi:10.3390/cancers17193133)

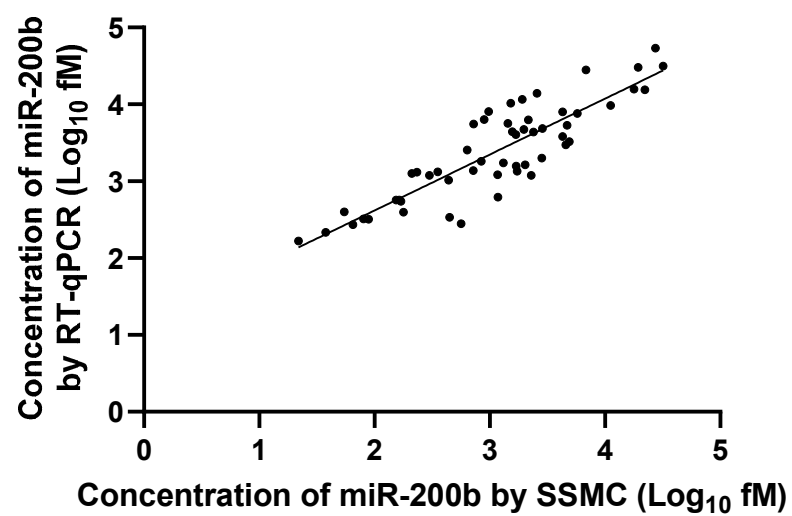

**Figure S1.** Linear regression analysis for concentrations of miR-200b between RT-qPCR and SSMC

Supplement: Supplementary file 1 [file cancers-17-03133-s001.zip › cancers-3857040-supplementary.pdf]
